# Association between primary caregiver type and mortality among Chinese older adults with disability: a prospective cohort study

**DOI:** 10.1186/s12877-021-02219-5

**Published:** 2021-04-21

**Authors:** Yangyujin Liu, Haoxue Li, Bei Wu, Xiaoting Liu, Honglin Chen, Hai-Yu Jin, Chenkai Wu

**Affiliations:** 1 Global Health Research Center, Duke Kunshan University, Academic Building 3038, No. 8 Duke Avenue, Kunshan, 215316 Jiangsu China; 2 Rory Meyers College of Nursing, New York University, New York, USA; 3 Department of Social Security & Risk Management, School of Public Affairs, Zhejiang University, Hangzhou, China; 4 Department of Social Work, Fudan University, Shanghai, China

**Keywords:** Informal caregiving, Caregiver, Unmet care needs, China, Death

## Abstract

**Background:**

Socio-demographic transitions have dramatically changed the traditional family care settings in China, caused unmet care needs among older adults. However, whether different primary caregiver types have different influences on disabled older adults’ health outcomes remain poorly understood. We aimed to examine the association between the type of primary caregiver (e.g., spouse and children) and death among community-dwelling Chinese older adults disabled in activities of daily living.

**Methods:**

We used data from Chinese Longitudinal Healthy Longevity Survey. The analytic sample comprised 4278 eligible adults aged ≥ 80 years. We classified primary caregiver type into five categories: spouse, son/daughter-in-law, daughter/son-in-law, grandchildren, and domestic helper. We used Cox regression model to examine the association between primary caregiver type and all-cause mortality. Covariates included age, sex, residence, years of education, co-residence status, financial independence, whether living with children, number of ADL disability, number of chronic conditions, and self-reported health, cognitive impairment, and caregiving quality.

**Results:**

Married older adults whose primary caregivers were son/daughter-in-law had a 38% higher hazard of death than those who had spouse as the primary caregiver. Married men who received care primarily from son/daughter-in-law or daughter/son-in-law had a 64 and 68% higher hazard of death, respectively, than those whose primary caregiver was spouse. The association between primary caregiver type and mortality among widowed older adults differed between urban and rural areas. Urban residents who had domestic helpers as the primary caregiver had an 16% lower hazard of death, while those living in rural areas had a 50% higher hazard of death, than those having son/daughter-in-law as the primary caregiver.

**Conclusions:**

The quality of care of the primary caregiver may be a risk factor for mortality of disabled older adults in China. Interventions are necessary for reducing unmet needs and managing care burden.

## Background

China is facing challenges brought by its rapid aging population, aging-associated disease burden and family structure changes. Its population aged ≥ 80 years is expected to increase fourfold from 22.6 million to 90.4 million between 2013 and 2050, while changes in family structure and traditional care arrangements have shifted the caregiving to older people [1, 2]. Several national surveys in China revealed that in 2011, around 10.4 to 13.3% of the Chinese adults aged ≥ 60 years had disability in activities of daily living (ADL), defined as the need for personal assistant in their basic ADL, including bathing, dressing, eating, transferring, toileting, and continence [3]. In addition, the prevalence of disability increases with advancing age, leading to a rapidly increasing needs for formal and informal care [4]. However, the existing healthcare system in China is not well-prepared for the increasing care needs among older adults in both urban and rural areas, and the home care support service provision is still in its infancy [2]. Moreover, although children, especially son and daughter-in-law, have traditionally played an essential role in the care for older adults in China, with social and family transitions, the pattern of primary caregivers for older people is rapidly changing. Yet, whether different primary caregiver types deliver the same quality of caregiving and have influences on disabled older adults’ health outcomes remain largely unknown.

Using data from a large longitudinal study in China, this study examined the association between primary caregiver type (e.g. spouse, children, and domestic helper) and mortality among disabled adults aged ≥ 80 years in China. We also examined whether the association between primary caregiver type and mortality differed by sex and residence location (urban or rural) of the disabled persons. Findings from this study may provide important information for the government to design and provide necessary support to the family caregivers for disabled older adults.

## Methods

### Data and study participants

Data were from the Chinese Longitudinal Healthy Longevity Survey (CLHLS), an ongoing prospective, longitudinal study with the largest sample of the oldest old (adults aged ≥ 80 years) in China. Half of the counties and cities in 22 of the 31 provinces in China (covering 85% of the population) were randomly selected through a multistage cluster sampling approach. A wide range of socio-demographic, lifestyle, and health measures were collected in the CLHLS. The baseline survey was conducted in 1998 and participants who were alive were re-interviewed in follow-up surveys conducted every 2 or 3 years. The CLHLS study was approved by the Ethical Review Committee at the Peking University, and written informed consent was obtained from all participants or their proxies. More details about the study design of the CLHLS have been documented elsewhere [5].

We used data from 2005, 2008, and 2011 waves of the CLHLS. We did not include data from 1998, 2000, and 2002 waves due to unavailability of information on primary caregiver type; we did not use data from 2014 wave because death information was only ascertained prior to 2014. A total of 18,278 individuals were surveyed in the 2005, 2008 or 2011 wave of the CLHLS. The analytic sample of this study comprised 4278 participants who: (i) were at least 80 years of age; (ii) reported sex; (iii) were community-dwelling (not nursing home residents); (iv) had at least one ADL disability (only persons who reported ADL disability were asked questions about primary caregiver); (v) had mortality data; (vi) were widowed or currently married (persons with other marital status were rare), (vii) whose primary caregivers were spouse, son/daughter-in-law, daughter/son-in-law, grandchildren, and domestic helper (others types including other relatives, neighbors, social services, and nobody were excluded due to small sample sizes). For participants who were surveyed in multiple waves, we used data from the first wave.

### Disability

In the CLHLS survey, the ADL disability in ADLs was assessed by asking the participants whether they could perform each task—dressing, bathing, eating, transferring, toileting, and continence—independently. Persons who answered “receiving assistance to one or more tasks” were considered being disabled.

### Caregiver type

The primary caregiver type was ascertained according to a question in the survey asking respondents: “Who is the primary helper when you need help with the six activities of daily living?” and classified into five categories: spouse, son/daughter-in-law, daughter/son-in-law, grandchildren, and domestic helper.

### Mortality

The outcome was all-cause mortality. Vital status and date of death were ascertained by the close family member or village doctor of the deceased participant. We calculated the survival time from the date of the first interview to the date of last interview or the death time, while we used the midpoint between the date of last interview and the date of second last interview as the end date of survival time for those who were lost to follow-up. The median follow-up time was 2.17 years.

### Covariates

Covariates included age, sex, residence (urban or rural), years of education, co-residence status, financial independence (yes or no), whether living with children, number of ADL disability (1 vs. 2+), number of chronic conditions, and self-reported health (very good, good, so-so, bad, and very bad), cognitive impairment (mini-mental state examination score < 18), and caregiving quality. All covariates were measured at baseline.

According to the CLHLS questionnaire, the self-reported chronic conditions included hypertension, diabetes mellitus, heart disease, respiratory disease, stroke/cerebrovascular disease, vision impairment, and Parkinson’s disease. We selected these variables due to two considerations: 1. To be consistent with the previous investigations using the CLHLS data [6]; and 2. The prevalence of each disease is not too low. Therefore, we decided to include self-reported hypertension, diabetes, heart disease, cerebrovascular disease, pulmonary disease, cancer, arthritis, and Parkinson’s disease in the present study.

### Statistical analysis

We first described the baseline socio-demographic and health characteristics of participants by primary caregiver type: spouse, son or daughter-in-law, daughter or son-in-law, grandchildren, and domestic helper. We used means and SDs for continuous variables and counts and percentages for categorical variables. We used analysis of variance and χ2 test for comparing continuous and categorical variables, respectively, across primary caregiver type. Then we described the relative frequency of each primary caregiver type by marital status (married and widowed).

**Fig. 1:**
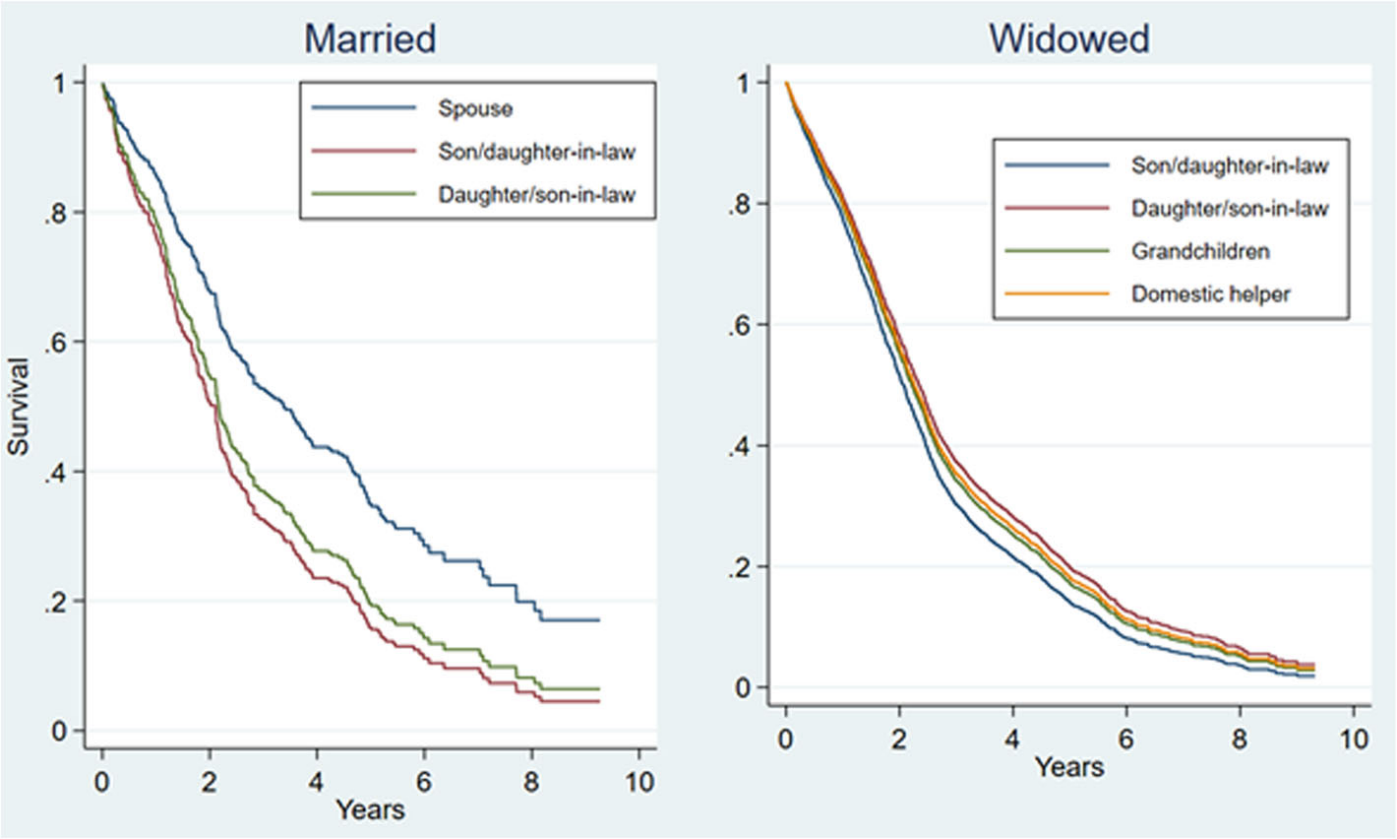
Survival rates by the kinship type of caregivers

We used Kaplan-Meier curves to show the unadjusted survival rates by the kinship type of caregivers (Fig. 1). We then examined the association between primary caregiver type and all-cause mortality using a Cox regression model. We first included age, sex, and number of ADL disability in the minimally adjusted models (Model 1), and then added residence, years of education, financial independence, living with children, self-rated health, number of chronic conditions, cognitive impaired, and caregiving quality. All variables, except for the “caregiving quality”, are for older adults who are being taken care of. The data of the “caregiving quality” variable is from the question asking whether the older adult think that helps in the six tasks he/she received could meet his/her needs in the CLHLS survey. Because caregiver type “spouse” is not applicable to widowed persons, analyses were conducted separately for married and widowed older adults (Model 2). For the married group, caregiver type “grandchildren” and “domestic helper” were excluded because of the small sample sizes (10 and 17, respectively). We used the interaction approach to examine whether the association between primary caregiver type and mortality differed by sex and residence location, respectively.

## Results

### Sample description

We described the frequency of caregiver type among married (8.4%) and widowed (91.6%) community-dwelling Chinese older adults with ADL disability (Table 1). For married persons, 50.6% had spouse as their primary caregiver, followed by son/daughter in law (29.4%), and daughter/son in law (13.1%). For widowed persons, the main caregiver type was son/daughter in law (62.0%), followed by daughter/son in law (21.8%), grandchildren (10.1%), and domestic helpers (6.1%).

**Table 1:** Caregiver type of married and widowed community-dwelling Chinese older adults with ADL disability

	Married (***N*** = 360)	Widowed (***N*** = 3918)	Total (***N*** = 4278)
	Count ()		
Spouse	182 (50.6)	–	182
Son or daughter-in-law	106 (29.4)	2430 (62.0)	2536
Daughter or son-in-law	47 (13.1)	852 (21.8)	899
Grandchildren	9 (2.5)	397 (10.1)	406
Domestic helper	16 (4.4)	239 (6.1)	255

*Note*: Other caregiver types (including other relatives, neighbors, social services, and nobody) were excluded due to small sample sizes

Older adults whose primary caregiver were spouse were more often male, younger, and less likely to be cognitively impaired (Table 2). In addition, compared with the older adults who had spouse as primary caregivers, those who had children or grandchildren as primary caregivers are older and more functionally (had difficulty in 3+ ADL) and cognitively impaired. The majority of the participants (85.5%) who had domestic helper as their primary caregiver were urban residents. Furthermore, older adults who had spouse and domestic helper as their primary caregivers reported more chronic conditions, and less likely to have a very good or good self-reported health than other groups.

**Table 2:** Baseline characteristics of community-dwelling Chinese older adults with ADL disability by caregiver type

	Spouse	Son or daughter-in-law	Daughter or son-in-law	Grand children	Domestic helper	*P*-value
	*N* = 182	*N* = 2536	*N* = 899	*N* = 406	*N* = 255	
	Count (%) or Means ± SD					
Age, years	90.1 ± 5.8	97.5 ± 5.7	97.7 ± 5.7	99.6 ± 4.8	97.3 ± 5.7	<.001
Female	40 (21.5)	1908 (75.2)	703 (78.2)	332 (81.8)	185 (72.6)	<.001
Urban residence	85 (45.7)	913 (36.0)	522 (58.1)	187 (46.1)	218 (85.5)	<.001
Education, years	1.9 ± 3.1	0.8 ± 2.1	1.0 ± 2.5	0.7 ± 2.1	2.7 ± 4.3	<.001
Financial independence	116 (62.4)	1947 (76.8)	684 (76.1)	312 (76.9)	204 (80.0)	<.001
Co-residence (vs. living alone)	184 (98.9)	2421 (95.5)	855 (95.1)	285 (94.8)	220 (86.3)	<.001
Living with children	81 (43.6)	2296 (89.7)	810 (90.2)	369 (91.1)	184 (72.2)	<.001
Hypertension	43 (23.1)	387 (15.3)	145 (16.1)	46 (11.3)	60 (23.5)	<.001
Diabetes	8 (4.3)	41 (1.6)	17 (1.9)	8 (2.0)	10 (3.9)	<.001
Heart disease	25 (13.4)	183 (7.2)	129 (14.4)	32 (7.9)	49 (19.2)	<.001
Cerebrovascular disease	40 (21.5)	217 (8.6)	76 (8.5)	29 (7.1)	327 (10.6)	<.001
Pulmonary disease	35 (18.8)	259 (10.2)	121 (13.5)	52 (12.8)	39 (15.3)	<.001
Cancer	3 (1.6)	9 (0.4)	7 (0.8)	3 (0.7)	1 (0.4)	<.001
Arthritis	41 (22.04)	463 (18.3)	166 (18.5)	100 (24.6)	69 (26.1)	<.001
Parkinson’s disease	4 (2.2)	21 (0.8)	8 (0.9)	2 (0.5)	1 (0.4)	<.001
Number of chronic diseases	1.1 (1.1)	0.6 (0.9)	0.7 (1.0)	0.7 (0.9)	1.0 (1.1)	<.001
Good/very good self-reported health	40 (21.5)	784 (30.9)	267 (29.7)	107 (26.4)	60 (23.5)	<.001
Number of ADL:						
Difficulty in 1 ADL	82 (44.1)	929 (36.6)	365 (40.6)	151 (37.2)	64 (25.1)	<.001
Difficulty in 2 ADL	27 (14.5)	386 (15.2)	116 (12.9)	53 (13.0)	24 (9.4)	
Difficulty in 3+ ADL	73 (41.4)	1221 (48.2)	418 (46.5)	206 (49.8)	167 (65.5)	
Cognitive impairment	78 (42.2)	1816 (71.8)	583 (64.9)	301 (74.3)	159 (62.4)	< 0.001

*Abbreviations*: *SD* Standard deviation, *ADL* Activity of daily living

Pulmonary disease: including bronchitis, emphysema, asthma, or pneumonia

Cognitive impairment: mini-mental state examination score < 18

### Association between primary caregiver type and mortality

The survival curves suggested that, among married older adults, those with spouse as the primary caregiver had the highest survival rate and those who had son/daughter-in-law as the primary caregiver had the lowest survival rate; we found the similar pattern among widowed ones (Fig. 1).

The Cox regression models showed that among older adults who were married, son or daughter-in-law as the primary caregiver was associated with a 38% (95% confidence interval [CI]: 4, 84%) higher hazard of death than spouse as the primary caregiver, adjusting for age, sex, and number of ADL disability (Model 1 in Table 3). The association remained significant after further adjustment of residence, education years, financial independence, self-rated health, number of chronic conditions, cognitive impairment, and caregiving quality (Model 2 in Table 3). Daughter or son-in-law as the primary caregiver was associated with a 33% higher hazard of death in the fully adjusted model than those who had spouse as the primary caregiver, although the association did not reach statistical significance (95% CI: 0.89, 2.10).

**Table 3:** Association between caregiver type and all-cause mortality among married and widowed Chinese older adults with ADL disability

		Model 1		Model 2	
	Incidence per 1000 PYs (95% CI)	HR (95% CI)	*P*-value	HR (95% CI)	*P*-value
	Married (N = 360)				
Spouse	198.9 (169.1, 233.9)	Ref.		Ref.	
Son/daughter-in-law	345.7 (279.2, 428.1)	1.38 (1.04, 1.84)	.022	1.41 (1.03, 1.91)	.029
Daughter/son-in-law	302.2 (213.7, 427.3)	1.27 (0.84, 1.93)	.263	1.33 (0.89, 2.10)	.194
	Widowed (N = 3918)				
Son/daughter-in-law	352.9 (336.9, 369.7)	Ref.		Ref.	
Daughter/son-in-law	294.0 (270.8, 319.2)	0.83 (0.76, 0.91)	<.001	0.88 (0.81, 0.96)	.003
Grandchildren	316.1 (280.1, 356.7)	0.85 (0.75, 0.97)	.013	0.86 (0.76, 0.97)	.015
Domestic helper	301.0 (255.7, 354.4)	0.81 (0.68, 0.95)	.013	0.85 (0.71, 1.01)	.070

*Abbreviations*: *PY* Person-year, *HR* Hazard ratio, *CI* Confidence interval, *ADL* Activity of daily living

Notes: Other caregiver types (including other relatives, neighbors, social services, and nobody) were excluded due to small sample sizes

Model 1: Adjusted for age, sex, and number of ADL disability

Model 2: Adjusted for validated age, sex, number of ADL disability, residence (urban vs. rural), education years, financial independence (yes vs. no), self-rated health (very good/good/so-so/bad/very bad), number of chronic conditions, cognitive impaired (MMSE< 18), and caregiving quality

Among the widowed older adults, daughter/son-in-law, grandchildren, and domestic helper as the primary caregiver was associated with a 17, 15, and 19% lower hazard of death, respectively, than son/daughter-in-law as the primary caregiver, adjusting for age, sex, and number of ADL disability (Model 1 in Table 3). These associations slightly attenuated but persisted to be significant in fully adjusted model (Model 2 in Table 3).

Among married older adults, compared with those whose primary caregiver was spouse, males who received care primarily from son/daughter-in-law or daughter/son-in-law had a 64 and 68% increase respectively in the hazard of death, whereas no significant results were found for their female counterparts (Table 4). Among those widowed older adults whose primary caregivers were domestic helpers, urban residents had an 16% decrease in the hazard of death, while the rural residents had a 50% increase in the hazard of death than those widowed older people whose primary caregivers were son/daughter-in-law (Table 5).

**Table 4:** Association between caregiver type and all-cause mortality among married and widowed Chinese older adults by sex

Sex of care receiver	Caregiver type	Multivariable adjusted with interaction term in sex and type		
		Count	HR (95% CI)	*P*-value
Female	Married (N = 89)			
	Spouse	37	Ref.	
	Son/daughter-in-law	28	0.90 (0.48, 1.76)	.763
	Daughter/son-in-law	19	0.95 (0.42, 2.04)	.907
	Widowed (*N* = 3076)			
	Son/daughter-in-law	1880	Ref.	
	Daughter/son-in-law	684	0.88 (0.79, 0.98)	.019
	Grandchildren	330	0.90 (0.78, 1.04)	.152
	Domestic helper	182	0.99 (0.82, 1.23)	.929
Male	Married (*N* = 271)			
	Spouse	145	Ref.	
	Son/daughter-in-law	78	1.64 (1.18, 2.30)	.004
	Daughter/son-in-law	28	1.68 (0.97, 2.92)	.065
	Widowed (*N* = 842)			
	Son/daughter-in-law	550	Ref.	
	Daughter/son-in-law	168	0.88 (0.70, 1.10)	.271
	Grandchildren	67	0.67 (0.48, 0.93)	.018
	Domestic helper	57	0.79 (0.54, 1.17)	.234

*Abbreviations*: *HR* Hazard ratio, *CI* Confidence interval, *ADL* Activity of daily living

Notes: Other caregiver types (including other relatives, neighbours, social services, and nobody) were excluded due to small sample sizes

Adjusted variables include age, number of ADL disability, residence (urban vs. rural), co-residence (living with children vs. not living with children), education years, financial independence (yes vs. no), self-rated health (very good/good/so-so/bad/very bad), number of chronic conditions, cognitive impaired (MMSE< 18), and caregiving quality

**Table 5:** Association between caregiver type and all-cause mortality among urban and rural Chinese older adults by residence

Residence of care receiver	Caregiver type	Multivariable adjusted with interaction term in residence and type		
		Count	HR (95% CI)	*P*-value
Urban	Married (*N* = 169)			
	Spouse	83	Ref.	
	Son/daughter-in-law	36	1.85 (1.14, 3.01)	.013
	Daughter/son-in-law	30	1.54 (0.86, 2.75)	.133
	Widowed (*N* = 1754)			
	Son/daughter-in-law	877	Ref.	
	Daughter/son-in-law	492	0.88 (0.77, 1.02)	.074
	Grandchildren	183	0.89 (0.72, 1.09)	.274
	Domestic helper	202	0.84 (0.68, 1.03)	.100
Rural	Married (*N* = 191)			
	Spouse	99	Ref.	
	Son/daughter-in-law	70	1.27 (0.87, 1.86)	.219
	Daughter/son-in-law	17	1.83 (0.92, 3.57)	.080
	Widowed (*N* = 2164)			
	Son/daughter-in-law	1553	Ref.	
	Daughter/son-in-law	360	0.87 (0.76, 1.00)	.046
	Grandchildren	214	0.84 (0.70, 1.00)	.055
	Domestic helper	37	1.50 (1.04, 2.18)	.031

Abbreviations: *HR* Hazard ratio, *CI* Confidence interval, *ADL* Activity of daily living

Notes: Other caregiver types (including other relatives, neighbours, social services, and nobody) were excluded due to small sample sizes

Adjusted variables include age, sex, number of ADL disability, co-residence (living with children vs. not living with children), education years, financial independence (yes vs. no), self-rated health (very good/good/so-so/bad/very bad), number of chronic conditions, cognitive impaired (MMSE< 18), and caregiving quality

## Discussion

Using data from a large, longitudinal study of older adults with ADL disability in China, we examined the patterns of primary caregiver among Chinese ADL disabled older adults aged 80 or above, and the association between the type of primary caregiver and mortality of these older adults. Regarding the logic of classifying caregiver types, it is worth noting that it might be a concern that different kinships with the care recipients can lead to different care quality and levels of stress perceived by care recipients. For instance, sons and daughters have blood relationships with the care recipients, while daughters-in-law and sons-in-law do not have this bond, which may cause more stress to the older adults and affect quality of care. However, in Chinese culture, sons and daughters-in-law (and also daughters and sons-in-law) typically provide care to older adults together. In addition, it is practically difficult to separate them into different groups due to small sample size. Therefore, we combined sons and daughters-in-law (and daughters and sons-in-law) into the same category. In addition, it should be noted that the caregiver may change over time (for example, spouse died and son took over the responsibility of caregiving), and potentially influence the study results. The primary reason that we only used the “baseline” information (2005, 2008, or 2011, depending on the timing of disability) was that the study sample was very old (every participant was disabled) and the majority died before having the chance of being surveyed again. Only a small portion (< 25%) of the study participants had follow-up data. Therefore, using a time-varying Cox model to account for repeated measures will unlikely to change the study results appreciably.

We found that apart from spouse, son/daughter in law was the second most common type of caregiver for Chinese ADL disabled older adults aged 80 years or above in both rural and urban areas. Apart from the older people’s choice and family members’ availabilities, some studies suggested that women have become more involved in workforce due to social and economic transitions [2, 7], thus it has become less common for daughters to work as caregivers for care-dependent parents in the family. The results also revealed that the primary caregiver type was associated with mortality of older adults with ADL disability in China, but the situation was different between the married and the widowed groups. When taken multiple social demographic and health related factors into consideration, married older adults who had daughter/son-in-law or son/daughter-in-law as primary caregiver had higher mortality rate compared with those who had spouse as primary caregiver. Regarding this finding, it should be noted that there are various mechanisms through which the survival rates differed by the kinship type of the caregivers. Firstly, different kinships between the caregiver and the care recipients might lead to differences in the level of psychological stress felt by the caregiver. For instance, a study conducted in Japan [8] suggested a potential survival “penalty” for older Japanese women who are cared for by their daughters-in-law, which may have further implications to other East Asian countries where daughters-in-law have played a central role in providing informal care for older people. In other words, the care from the in-laws might cause more stress than the care from the older adults’ own children. In addition, the amount of time spent on care from a son or daughter might be shorter compared to care from a spouse, which can affect the care outcome. Therefore, these results should be interpreted with the quality of care the caregivers provide, which can vary considerably among different types of caregivers due to individual, social, and manifold factors.

It is noticeable that urban married older adults who had son/daughter-in-law as primary caregiver had significant increase in mortality rate. Some studies suggest that stress of caregivers can affect the care quality and lead to negative health outcomes to the care-dependent older people [9–13]. This issue seems to be particularly prominent for Asian female family caregivers. For instance, a study [13] suggested that the stress from multiple family roles of women in a multi-generational family in Asian culture could contribute to higher risk of certain diseases. The sons/daughter-in-law need to attend both parents and their own nuclear families apart from their career duties, while the faster-paced urban life can intensify the pressure to these caregivers, reduce their time and energy for care giving, thus deteriorating the quality of their care. In addition, among married older adults, compared with those whose primary caregiver was spouse, males who received care primarily from son/daughter-in-law or daughter/son-in-law had increase in the hazard of death, which were opposite from the female participants. This result suggests a sex difference in the care outcome of the older adults with ADL disability, in which female older adults outperformed the male older adults. This finding corresponds with previous findings suggesting that older women are more resilient in adversities, partly due to their access to resources that help them overcome hardships in later life, including social support [14, 15].

Indeed, women have lower mortality rates and longer life expectancy than men, and some researchers argue that it is because women have biological advantages than men in dealing with certain diseases [16–18], while others think it relates to health behaviours: for instance, there is a higher prevalence of smoking behaviour among men [19–21]. However, while men are more vulnerable to major life-threatening chronic diseases, including coronary heart disease, cancer, kidney disease, and atherosclerosis, women are more prone to a number of not life-threatening chronic conditions, including arthritis, migraine headaches, hearing and vision loss [18, 19]. Therefore, it is important to note that longer lifespan of the female older people does not indicate that their quality of life is better, and the quality of care they receive from the primary caregivers need to be further evaluated for improvement.

Moreover, it should be noted that for those older adults whose primary caregivers were domestic helpers, the urban residents had a 18% decrease in the hazard of death comparing with those whose primary caregivers were son/daughter-in-law, while the number jumped to 48% for the rural adults. This may require further investigation for the reason behind. As suggested by some studies, socioeconomic characteristics have influence on health outcomes [22], and generally rural areas have less care resources, quality, and personnel, which can negatively affect the care quality of the older adults with ADL disability.

Findings of this study highlight the needs to develop interventions to support the family care for the Chinese older adults with ADL disability and their family caregivers. On the one hand, it is important to train the family caregivers to fully identify the needs of the older adults, and provide necessary care and support in a timely manner [23, 24]. On the other hand, community services (such as respite care) should be strengthened to support family caregivers, and resources need to be provided to reduce their caregiving burden, especially for those who are experiencing stress from both work and family duties. The long-term care insurance should be paid to the informal caregiver and reduce their financial burden.

We acknowledge several limitations. First, the oldest old sample has a problem of selection bias as more female are widowed than male, while the sample size is unbalanced between the married and widowed older adults with a relatively small sample size of the married older adults. In addition, the sample size of rural older adults who had domestic helper as the primary caregiver is relatively small. Second, some participants had family members as the proxies to answer the survey questions, who may have different attitudes toward perceived unmet needs for services [25–27], thus the answers given by representatives may have underestimated the situation of unmet care needs of the older people. Third, due to the lack of data on caregiver factors, we used the type of primary caregivers as a proxy to measure the care quality, and we did not include information on secondary caregivers in this analysis. It would be more ideal to use data that directly measure the quality of care these primary caregivers provide for analysis. These limitations should be well considered for future studies, especially to include variables on caregiver factors if data become available. Lastly, it should be noted that the death certification is not verified by vital statistics in this study.

In sum, family caregivers’ support and involvement are essential to guarantee the basic care for the care-dependent older people in China. Family members and domestic helpers who are providing care to the ADL disabled older adults should obtain training on identifying care needs and arranging caregiving activities. Furthermore, male and female older adults with ADL disabilities may have different care needs, thus require different support and resources, which deserves further research and understanding. Efforts should also be made to evaluate the potential burden and stress of the family caregivers related to the caregiving role. In addition, counselling and other supportive services should be provided to family caregivers, to prevent unmet care needs worsening into more adverse consequences to the older care recipients.

## Data Availability

The datasets analyzed during the current study are available in the Chinese Longitudinal Healthy Longevity Survey (CLHLS), Community Datasets, 1998–2014 (ICPSR 37227), https://www.icpsr.umich.edu/web/NACDA/studies/37227/versions/V1

## Abbreviations

ADL: Activities of daily living
CI: Confidence interval
CLHLS: Chinese Longitudinal Healthy Longevity Study
HR: Hazard ratio
MMSE: Mini Mental State Examination
PY: Person-year
SD: Standard deviation
